# Effect of the Green/Blue Flicker Matrix for P300-Based Brain–Computer Interface: An EEG–fMRI Study

**DOI:** 10.3389/fneur.2012.00113

**Published:** 2012-07-11

**Authors:** Shiro Ikegami, Kouji Takano, Makoto Wada, Naokatsu Saeki, Kenji Kansaku

**Affiliations:** ^1^Systems Neuroscience Section, Department of Rehabilitation for Brain Functions, Research Institute of National Rehabilitation Center for Persons with DisabilitiesTokorozawa, Japan; ^2^Department of Neurological Surgery, Chiba University Graduate School of MedicineChiba, Japan

**Keywords:** BCI, BMI, P300, color, EEG–fMRI

## Abstract

The visual P300-brain–computer interface, a popular system for EEG-based BCI, utilizes the P300 event-related potential to select an icon arranged in a flicker matrix. In the conventional P300-BCI speller paradigm, white/gray luminance intensification of each row/column in the matrix is used. In an earlier study, we applied green/blue luminance and chromatic change in the P300-BCI system and reported that this luminance and chromatic flicker matrix was associated with better performance and greater subject comfort compared with the conventional white/gray luminance flicker matrix. In this study, we used simultaneous EEG-functional magnetic resonance imaging (fMRI) recordings to identify brain areas that were more enhanced in the green/blue flicker matrix than in the white/gray flicker matrix, as these may highlight areas devoted to improved P300-BCI performance. The peak of the positive wave in the EEG data was detected under both conditions, and the peak amplitudes were larger at the parietal and occipital electrodes, particularly in the late components, under the green/blue condition than under the white/gray condition. fMRI data showed activation in the bilateral parietal and occipital cortices, and these areas, particularly those in the right hemisphere, were more activated under the green/blue condition than under the white/gray condition. The parietal and occipital regions more involved in the green/blue condition were part of the areas devoted to conventional P300s. These results suggest that the green/blue flicker matrix was useful for enhancing the so-called P300 responses.

## Introduction

The brain–computer interface (BCI) or brain–machine interface (BMI) is an interface technology that utilizes neurophysiological signals from the brain to control external machines or computers, and has become widespread in this decade due to technical and mechanical improvements (Wolpaw et al., 2002; Birbaumer and Cohen, 2007; Kansaku, 2011). One research approach to BCIs relies on electrical signals recorded from the cortical surface [electrocorticograph (ECoG)] or directly from the neuron (unit recording); this approach can be categorized as invasive BCI because it requires neurosurgery (Kennedy et al., 2000; Hochberg et al., 2006; Yanagisawa et al., 2009; Brunner et al., 2011; Krusienski and Shih, 2011). Another approach utilizes neurophysiological signals from the brain that are accessed without surgery, which is called non-invasive BCI. The so-called P300 speller (Farwell and Donchin, 1988), one popular non-invasive BCI system, utilizes the visual P300 event-related potential (ERP) from scalp EEG electrodes. In the conventional P300 speller paradigm, each row/column of the matrix intensified with luminance change in a random sequence. Target stimuli are as rare stimuli used (i.e., the oddball paradigm) to elicit P300 responses. The recorded EEG signals are classified to detect the target at the intersection of the predicted row and column. Recently, several P300-BCI systems were developed and tested on individuals with amyotrophic lateral sclerosis, cervical spinal cord injury, and other disorders (Piccione et al., 2006; Sellers and Donchin, 2006; Hoffmann et al., 2008; Nijboer et al., 2008; Ikegami et al., 2011).

In the P300-BCI system, the parietal and occipital electrodes are effective (Krusienski et al., 2008; Rakotomamonjy and Guigue, 2008; Cecotti et al., 2011), and a configuration with parietal and occipital electrodes is commonly used (Nijboer et al., 2008; Takano et al., 2009b, 2011; Kansaku et al., 2010; Townsend et al., 2010; Ikegami et al., 2011). The importance of the parietal and occipital electrodes for achieving better performance of the P300-BCI approach has also been reported (Bianchi et al., 2010; Brunner et al., 2010; Treder and Blankertz, 2010). In recent invasive P300-BCI studies, the ECoG electrodes were placed around the parietal and occipital lobes, and successful operation of the visual P300 speller was reported (Brunner et al., 2011; Krusienski and Shih, 2011).

If the chosen BCI communication system results in a rate of correct responses that exceeds 70%, it has potential for practical use as a BCI system in people with disabilities (Kubler et al., 2001; Sellers et al., 2006; Kubler and Birbaumer, 2008; Nijboer et al., 2008). Several methods of classification have been investigated to improve the accuracy of P300-BCI systems (Donchin et al., 2000; Kaper et al., 2004; Krusienski et al., 2006; Bashashati et al., 2007; Hoffmann et al., 2008). Other studies have investigated effective visual presentation, such as matrix size (Sellers et al., 2006), inter-stimulus intervals (Allison and Pineda, 2006; Sellers et al., 2006), flash patterns (Guger et al., 2009; Townsend et al., 2010), background colors (Salvaris and Sepulveda, 2009), and the presentation of moving targets (Guo et al., 2008; Martens et al., 2009; Treder and Blankertz, 2010). These studies succeeded in improving the performance of BCI systems. We prepared a green/blue luminance and chromatic flicker matrix as the visual stimuli, following a previous study of patients with photosensitive epilepsy (Parra et al., 2007).

Parra et al. (2007) evaluated the safety of chromatic combinations for patients with photosensitive epilepsy. Five single-color stimuli (white, blue, red, yellow, and green) and four alternating-color stimuli (blue/red, red/green, green/blue, and blue/yellow with equal luminance) of four frequencies (10, 15, 20, and 30 Hz) were used as the visual stimuli. Flickering stimuli with higher frequencies, particularly those >20 Hz, are potentially provocative under white stimulation. The 15-Hz blue/red flicker was the most provocative under the alternating-color stimulation condition, as suggested by the Pokemon incidence (Ishida et al., 1998; Takahashi and Tsukahara, 1998). Notably, the green/blue chromatic flicker emerged as the safest and evoked the lowest rates of EEG spikes. We showed that the green/blue flicker matrix was associated with greater subjective feelings of comfort and superior performance than was the traditional white/gray flicker matrix (Takano et al., 2009a,b). The BCI system has been successfully used by individuals with cervical spinal cord injuries (Ikegami et al., 2011).

Although these researchers previously found improved BCI performance, the underlying neuronal mechanisms are not yet fully understood. Recent visual ERP-based BCI studies on eye gaze revealed neuronal responses in addition to P300, including early components of visual evoked potentials, which influenced the P300 speller performance (Brunner et al., 2010; Treder and Blankertz, 2010). In our earlier study using chromatic change, we showed enhanced P300-BCI performance. However, whether the P300-BCI improvement is caused by the so-called P300 response itself or by additional phenomena is unknown.

While EEG provides the temporal fidelity necessary to study ERPs, it lacks the capacity to localize the subcortical structures that elicit these ERPs. Functional magnetic resonance imaging (fMRI) not only has higher spatial resolution, but also has this capacity to localize candidate areas that elicit these ERPs in the whole brain. In this study, we used simultaneous EEG–fMRI to record brain signals during the P300-BCI paradigm using the green/blue flicker matrix and compared the findings with those for to the white/gray flicker matrix. We hypothesized that the green/blue flicker matrix would show enhanced activation within the parietal and occipital areas, which are known to be involved in the conventional P300 (relative to the white/gray flicker matrix), which would explain why the green/blue flicker matrix improves P300-BCI performance.

## Materials and Methods

### Participants

Twelve healthy volunteers (aged 20–36 years; all men) participated in this experiment. All subjects were neurologically normal and right-handed (min/mean/max = 0.8/0.87/1) according to the Edinburgh Inventory (Oldfield, 1971). No subject had a history of neurological disorders such as epilepsy or brain injury. This study received approval from the Institutional Review Board of the National Rehabilitation Center for Persons with Disabilities, Tokorozawa, Japan. All subjects provided written informed consent according to institutional guidelines.

### Tasks

**Figure 1:**
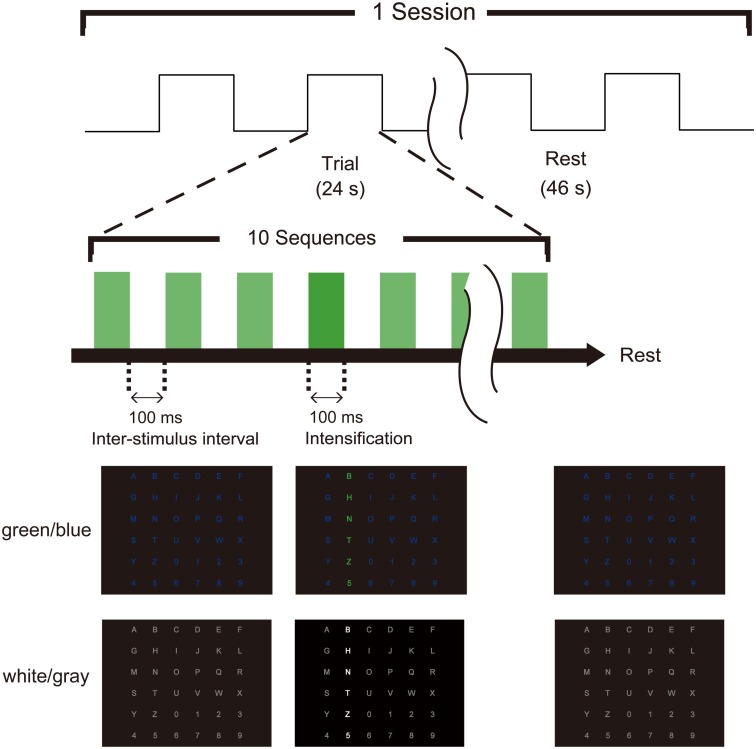
**Task timing and visual stimuli for the P300-brain–computer interface (BCI)**. Two types of flicker matrices were presented (green/blue and white/gray). The row or column was intensified randomly. The stimulus onset asynchrony was 200 ms, consisting of 100 ms of intensification and a 100-ms inter-stimulus interval. Ten sequences constituted a trial, and the subject focused on one target icon during each trial. EEG data were collected with the trigger of intensification, and fMRI data were recorded simultaneously.

During the simultaneous recordings of EEG and fMRI, each participant lay supine on the stretcher of an MR scanner with his head held in a vacuum cushion inside the head coil to restrict head motion. The visual stimuli (Figure 1) were projected onto a screen located at the rear of the scanner, and the subjects viewed the matrix through a mirror mounted on the head coil. Just before the experiment, each participant confirmed that they could see the visual stimuli completely. The stimulus was presented and synchronized with the MR scanner using Presentation^®^ (Neurobehavioral Systems, San Francisco, CA, USA). We applied simultaneous recordings of EEG and fMRI because they allow us to provide fine temporal and spatial resolution, although similar results could have been obtained through a more simple experimental setup (i.e., separately recording EEG and fMRI).

We used two types (green/blue luminance and chromatic condition and white/gray luminance condition; Figure 1) of visual-flicker stimuli in a 6 × 6 alphabet flicker matrix modified from the “P300 speller” (Farwell and Donchin, 1988; Donchin et al., 2000). We prepared a white (20 cd/cm)/gray (6.5 cd/cm) matrix for the white/gray condition, and a green (20 cd/cm)/blue (6.5 cd/cm) matrix for the green/blue condition, and luminance was measured using a chromatic meter (CS-200, Konica Minolta Sensing, Inc., Osaka, Japan) on the computer screen as we did in a previous study (Takano et al., 2009b). We used 100 ms of intensification and a 100-ms inter-stimulus interval because recent visual P300-BCI studies have usually used 125–300 ms for stimulus onset asynchrony (SOA) to facilitate rapid communication (Sellers et al., 2006; Takano et al., 2009b, 2011; Kansaku et al., 2010; Townsend et al., 2010; Ikegami et al., 2011; Pires et al., 2011). Each row/column of the matrix was intensified randomly. One complete cycle of six rows and six columns constituted a sequence (2 target stimuli and 10 non-target stimuli), and 10 sequences constituted a trial. During a trial, the participants were asked to focus on one of the icons as the target in the matrix. The target stimuli were presented as rare stimuli to elicit P300 responses (i.e., the oddball paradigm); 20 target stimuli and 100 non-target stimuli were presented in one trial. We conducted 21 trials during three separate sessions under both the green/blue and white/gray conditions and simultaneously recorded scalp EEG signals and blood-oxygen-level-dependent fMRI signals during each session. In the first session, the six-character alphanumeric code “ATI1Q9,” which was located in different rows and columns in the matrix, was used as a target. The eight-character code NRCDP300 was used for the second session, and the seven-character code BMITEST was used for the third session. Feedback was not presented. The order of the experimental conditions (green/blue or white/gray) was counterbalanced among subjects.

### EEG acquisition

**Figure 2:**
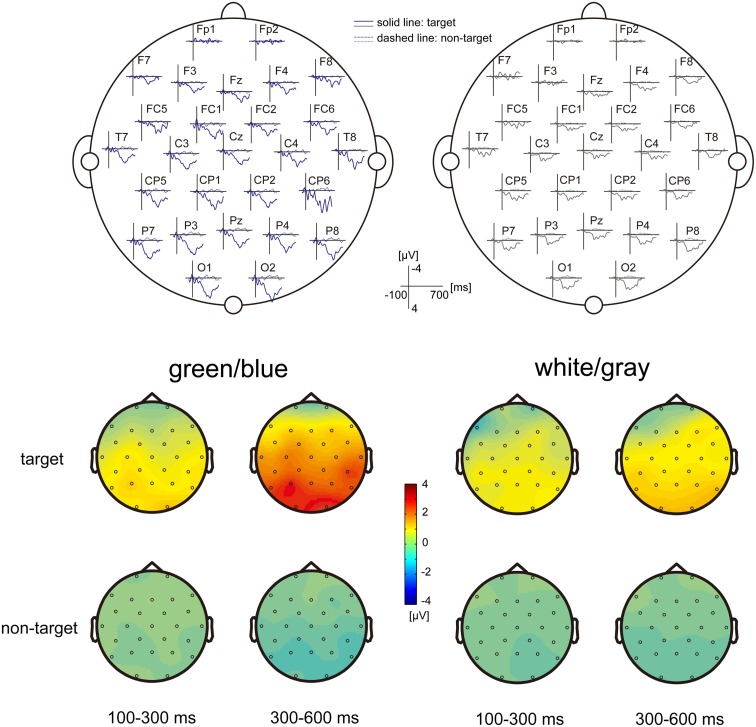
**EEG scalp topography**. (Upper) The grand-average scalp topography of the event-related potentials (ERPs) under the green/blue and white/gray conditions (*n* = 9). Dashed lines indicate non-target ERPs, and solid lines indicate the targeted ERPs. The green/blue or white/gray condition is shown in blue or gray, respectively. The P300 components are shown under both conditions, particularly over the parietal and occipital areas. (Lower) The scalp distribution of the early component (100–300 ms), and the late component (300–600 ms). The difference between the target and non-target was prominent in the late component under the green/blue condition and was located at the parietal and occipital sites.

**Figure 3:**
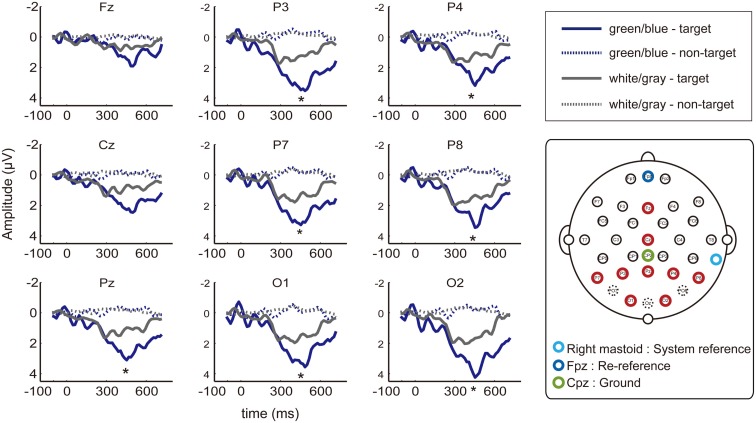
**EEG waveforms at nine electrodes**. Event-related potentials (ERPs) from the nine of the electrodes placed at 27 locations (Fz, Cz, P7, P3, Pz, P4, P8, O1, and O2; red circle) that have been used in previous P300-BCI studies. Dashed lines indicate non-target ERPs, and solid lines indicate the target. The green/blue or white/gray condition is shown in blue or gray, respectively. Asterisks indicate significantly larger amplitudes under the green/blue condition than under the white/gray condition (P7, P3, Pz, P4, P8, O1, and O2; *p* < 0.05, paired *t*-test).

In this study, we simultaneously recorded scalp EEG and blood-oxygen-level-dependent fMRI signals to measure brain signals from individual brains during the P300 speller tasks. We used an fMRI-compatible full-band DC-EEG system with a 32-channel EEG cap (NEURO PRAX MR, NeuroConn, GmbH, Grenzhammer, Germany) and recorded 27-channels of EEG signals (Fp1, Fp2, F7, F3, Fz, F4, F8, Fc5, Fc1, Fc2, Fc6, T3, C3, Cz, C4, T4, Cp5, Cp1, Cp2, Cp6, T5, P3, Pz, P4, T6, O1, and O2 according to the extended 10–20 international system, Figures 2 and 3) during the P300 speller tasks that were completed inside the MR scanner. Vertical eye movements were recorded with two electrodes placed above and below the right eye, and an electrocardiogram was also recorded using two left anterior chest electrodes. All channels were referenced to the right mastoid and grounded to CPz. The continuous EEG signals were digitized at 4000 Hz, processed with an online average subtraction method to correct for gradient artifacts (Mandelkow et al., 2006; Goncalves et al., 2007; Grouiller et al., 2007), and stored.

### EEG analysis

The recorded EEG data were used to evaluate the elicited ERP waveform. For offline ERP analysis, the 27-channels of recorded EEG data were re-referred to the Fpz (average of Fp1 and Fp2) and down-sampled to 64 Hz for analysis, as previously performed (Takano et al., 2009b, 2011; Kansaku et al., 2010; Ikegami et al., 2011). We conducted 21 trials under both the white/gray and green/blue conditions; thus, we collected 420 target responses and 2100 non-target responses from each subject under each condition.

An 800 ms sector of EEG data was segmented according to the timing of the onset of intensification. The first 100 ms occurring just prior to the onset of intensification was used for baseline correction. After artifact rejection, we depicted the grand-average scalp topography of the ERPs under the green/blue and white/gray conditions.

We focused on data from 9 of the 27 channel EEG (Fz, Cz, P7, P3, Pz, P4, P8, O1, and O2; Figure 3, red circle), which was similar to the electrode configuration used in previous P300-BCI research (Fz, Cz, P3, Pz, P4, PO7, Oz, and PO8; Krusienski et al., 2008; Takano et al., 2009b, 2011; Kansaku et al., 2010; Ikegami et al., 2011). The effects of flicker matrix condition (green/blue vs. white/gray) and electrode location [center (Fz, Cz, and Pz) vs. left (P7, P3, and O1) vs. right (P4, P8, and O2)] in the positive peak amplitude (baseline-to-peak) were examined using two-way repeated-measure analysis of variance (ANOVA), with the Tukey–Kramer test as a *post hoc* test. Additionally, the positive peak amplitude (baseline-to-peak) differences between green/blue and white/gray at each of nine electrodes were tested using a paired *t*-test.

### fMRI scanning

Because fMRI has better spatial resolution than EEG, we also recorded fMRI signals to detect brain areas that were additionally enhanced under the green/blue flicker matrix condition compared with the white/gray flicker matrix. Functional MR images [gradient echo T2* weighted echo-planar imaging sequences (EPI); TR/TE, 2900/40 ms; flip angle, 85°; slice thickness/gap, 5/2 mm; FOV, 220 mm; matrix, 64 × 64; 20 slices] were collected on a 1.5 T MR scanner (Exelart, Toshiba, Tokyo, Japan) during the sessions. The total of six sessions for each subject consisted of 1,194 scans, and the first three scans of each session were discarded due to T1 equilibration effects.

### fMRI analysis

We used SPM8 (Wellcome Department of Imaging Neuroscience, Institute of Neurology, University College London, UK) operating under a Matlab 2007a environment to preprocess and analyze the fMRI data. The realigned images were normalized to a Montreal Neurological Institute (MNI) EPI template and smoothed with an 8-mm full-width at half-maximum Gaussian kernel. A high-pass filter with a cut-off period of 128 s was set to remove low-frequency noise, and an autoregressive (order one) model was used to correct for short-range serial correlations. First, we conducted a fixed-effects analysis for each participant to obtain a contrast image for each condition. The contrast images of the 12 subjects were then used for the random-effects analysis. We first simply compared the difference in activation between conditions. Then we applied an inclusive mask (masked by activated regions of the green/blue condition) to confirm if predominant deactivations existed. We specifically focused on the occipital and parietal areas based on previous studies (Clark et al., 2000; Bledowski et al., 2004a,b; Krusienski et al., 2008; Rakotomamonjy and Guigue, 2008; Bianchi et al., 2010; Brunner et al., 2010; Treder and Blankertz, 2010; Cecotti et al., 2011), and threshold of random effect SPM{t} maps were set at an uncorrected *p* < 0.001 for each condition and an uncorrected *p* < 0.005 to compare the white/gray condition to the green/blue condition. The MNI coordinates were converted to Talairach coordinates (Talairach and Tournoux, 1988) using the non-linear transformations suggested by Brett (http://imaging.mrc-cbu.cam.ac.uk/imaging/MniTalairach). The corresponding Brodmann areas were first roughly approximated using Talairach Daemon (Lancaster et al., 2000) and then determined using the atlas of Talairach and Tournoux (1988).

## Results

### EEG

We used simultaneous EEG–fMRI recordings to investigate enhanced brain areas in the green/blue flicker matrix relative to the white/gray flicker matrix. The recorded EEG data would be most beneficial if they could be used for evaluating P300-BCI performance. However, the residual artifacts, particularly gradient artifacts (6.9 Hz), were so severe that we had to discard the data of three of the 12 subjects. Additionally, artifacts remained in the EEG data from nine subjects, so we manually removed 130 of 252 trial data under the green/blue flicker condition and 135 of 252 trial data under the white/gray flicker condition. In each channel, EEG data from 122 trials (2440 target responses and 12200 non-target responses) for the green/blue condition and data from 117 trials (2340 target responses and 11700 non-target responses) for the white/gray condition were averaged. Thus, the number of acquired trial data from each of the nine subjects was insufficient to evaluate P300-BCI task performance. Similarly it was insufficient to apply other analyses (e.g., source localization) to associate the fMRI and EEG results. Therefore, we simply evaluated the evoked response offline.

Figure 2 shows the grand-average scalp topography of the ERPs under the green/blue and white/gray conditions (*n* = 9). Positive peaks were detected between 300 and 600 ms after stimulus onset under both conditions, particularly over the parietal and occipital areas. The scalp distribution of the early component (100–300 ms) and the late component (300–600 ms) are shown at the bottom of Figure 2. The scalp distributions are shown separately for events in which the target icon intensified and events in which the non-target icon intensified. Note that activation in the parietal and occipital areas was more prominent in the late component (300–600 ms), when the target icon intensified.

We recorded data from 27 EEG channels, and we further focused on the nine electrodes (Fz, Cz, P7, P3, Pz, P4, P8, O1, and O2; red circle in Figure 3) that have been frequently used in previous P300-BCI studies (Krusienski et al., 2008; Takano et al., 2009b, 2011; Kansaku et al., 2010; Ikegami et al., 2011). The peak amplitudes of the ERPs between 300–600 ms after stimulus onset were analyzed. We used a two-way repeated-measure ANOVA to examine the main effects of condition (green/blue vs. white/gray) and electrode location [center (Fz, Cz, and Pz) vs. left (P7, P3, and O1) vs. right (P4, P8, and O2)]. ANOVA revealed effects of flicker matrix condition [*F*(1,48) = 13.86, *p* < 0.01] and electrode location [*F*(2,48) = 4.17, *p* < 0.05]. There was no significant interaction between the two variables [*F*(2,48) = 1.24, *p* = 0.29]. *Post hoc* testing revealed that locating the electrode on the right resulted in significantly greater amplitude compared with the center location (Tukey–Kramer test, *p* < 0.05). We further analyzed the peak amplitudes at nine electrodes using a paired *t*-test, and a significantly larger amplitude under the green/blue than under the white/gray condition was observed for seven of nine selected electrodes (P7, P3, Pz, P4, P8, O1, and O2; *p* < 0.05, paired *t*-test).

### Functional magnetic resonance imaging

**Figure 4:**
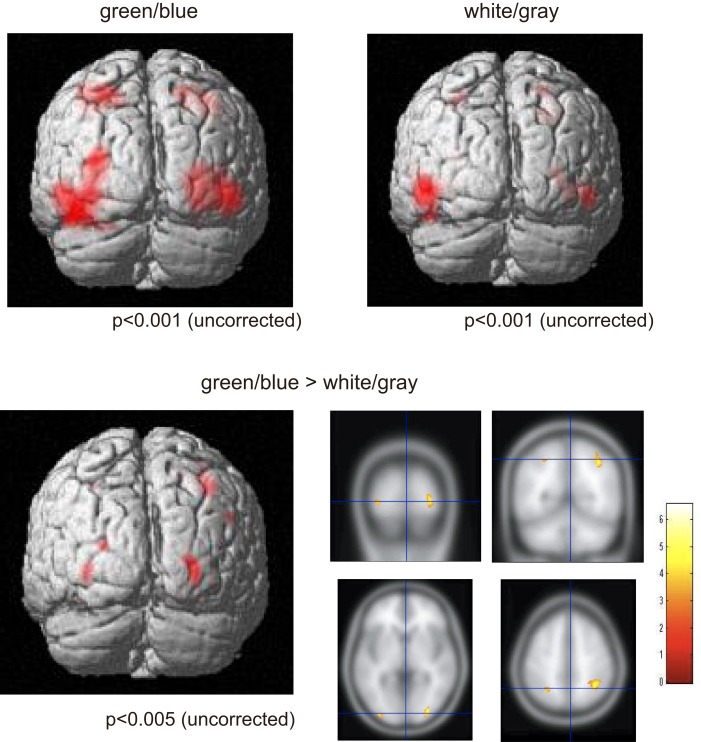
**Functional magnetic resonance imaging activation**. (Upper) fMRI activities under both the green/blue, and white/gray, conditions. The bilateral parietal and occipital areas were activated under both the green/blue and white/gray conditions (*P* < 0.001, uncorrected). (Lower) More significant activation in the parietal and occipital cortices (right > left) was evident under the green/blue condition than under the white/gray condition (*P* < 0.005, uncorrected). Note that the activation foci detected by the fMRI analyses were located close to the areas in which the peak ERP amplitudes in response to the target stimuli were significantly higher under the green/blue than under the white/gray condition.

We used the fMRI data to evaluate the spatial distribution of the activations. Activities under the green/blue and white/gray conditions were found in the bilateral occipital and parietal areas under both conditions (*P* < 0.001, uncorrected; Figure 4). The results did not change with or without an inclusive mask (masked by activated regions of the green/blue condition). We specifically focused on the occipital and parietal areas because in the EEG results we found a significantly larger amplitude under the green/blue than under the white/gray condition in these areas as described above. The Talairach coordinates of the activated regions are presented in Table 1. Note that the activation foci detected by the fMRI analyses were mainly located close to the areas in which recordings are usually performed during P300-BCI tasks.

**Table 1:** **Brain regions activated during P300-brain–computer interface (BCI) operations**.

Side	Brain region	Brodmann area	Cluster size	*Z*-value	Talairach coordinates		
					*x*	*y*	*z*
**GREEN/BLUE**							
Left	Superior parietal lobule	7	915	5.33	−28	−60	45
Right	Fusiform gyrus	37	1,778	5.20	41	−57	−6
Right	Inferior parietal lobule	7	734	5.18	33	−57	46
Left	Posterior cingulate	30	2,528	4.92	−29	−74	11
Left	Precentral gyrus	4	332	3.99	−44	−12	53
**WHITE/GRAY**							
Left	Middle temporal gyrus	37	1,101	4.71	−44	−63	−1
Right	Middle temporal gyrus	37	500	4.27	41	−58	−4
Right	Cuneus	19	22	3.74	20	−82	36
Right	Precuneus	7	46	3.67	20	−68	50
Right	Precentral gyrus	6	42	3.66	24	−16	52
Left	Lingual gyrus	19	23	3.29	−29	−76	−3
**GREEN/BLUE > WHITE/GRAY**							
Right	Inferior parietal lobule	40	219	4.10	32	−53	41
Right	Middle occipital gyrus	18	117	3.57	27	−84	−1
Right	Middle temporal gyrus	39	25	3.35	45	−61	26
Left	Middle occipital gyrus	18	19	3.34	−24	−90	8
Left	Superior frontal gyrus	6	18	3.16	−17	19	63
Left	Middle occipital gyrus	18	42	3.15	−33	−83	−8
Left	Insula	13	41	3.05	−34	16	18
Left	Superior parietal lobule	7	20	3.03	−28	−58	43
Right	Medial frontal gyrus	6	22	2.91	17	−18	55
Right	Anterior cingulate	24	22	2.87	12	21	21
**GREEN/BLUE < WHITE/GRAY**							
Left	Superior temporal gyrus	41	100	3.54	−40	−40	11

We compared responses under the green/blue condition to those under the white/gray condition to identify brain regions enhanced by chromatic change. The parietal and occipital cortices (right > left) were more activated under the green/blue than under the white/gray condition (*P* < 0.005, uncorrected; Figure 4, lower). More significant activation was found in the right inferior parietal lobule, middle occipital gyrus, middle temporal gyrus (BA40, BA18, and BA39 respectively), and left middle occipital gyrus (BA 18; Table 1). The most significant activation was observed in the right inferior parietal lobule (*x* = 32, *y* = −53, *z* = 41, Talairach coordinates, Table 1). In opposite contrasts, the superior temporal gyrus was more activated under the white/gray than under the green/blue condition (*P* < 0.005, uncorrected).

## Discussion

We used simultaneous EEG and fMRI recordings to investigate brain areas that were additionally enhanced in the green/blue flicker matrix compared with the white/gray flicker matrix, as these may highlight areas devoted to improved P300-BCI performance. According to the EEG data, the peak latency of the positive wave was detected between 300 and 600 ms, i.e., the late component, and the peak amplitudes were higher under the green/blue condition than under the white/gray condition at the parietal and occipital electrodes, especially on the right side. The fMRI data showed activation in the bilateral parietal and occipital cortices and brain areas, particularly in the right hemisphere, showed greater activation under the green/blue than under the white/gray condition.

### Brain areas additionally involved in the green/blue condition

Our results suggest the importance of the parietal and occipital areas in P300-BCI phenomena. Additional fMRI activation was observed in the bilateral superior parietal lobule under the green/blue condition, an area known to be activated during target-detection tasks that is linked to the location to which attention was directed before presentation of the visual target (Corbetta et al., 2000).

Our study further showed that the peak amplitudes were higher under the green/blue condition than under the white/gray condition at the parietal and occipital electrodes, especially on the right side, and fMRI activation in the bilateral parietal and occipital areas, particularly in the right hemisphere, was more highly activated under the green/blue condition than under the white/gray condition. The right hemisphere may be more involved in color detection; indeed, a recent psychophysical study suggested the superiority of the right hemisphere for detecting color (Sasaki et al., 2007). It is noteworthy that our study showed that peak EEG amplitudes were higher under the green/blue than under the white/gray condition at the parietal and occipital electrodes. These results suggest the ease with which changes can be detected under the green/blue flicker condition, which is consistent with Polich’s observation that the P300 amplitude is smaller in response to more difficult tasks than to easier tasks (Polich, 1987). Taken together, the green/blue color visual stimuli may have allowed the participants to detect the changes more easily, probably with the help of their right brain hemisphere.

### Brain areas for conventional P300s and P300-BCI

P300 tasks have been repeatedly used, and the neuronal bases have been investigated (Smith et al., 1990; Clark et al., 2000; Dien et al., 2003; Bledowski et al., 2004a,b); e.g., Bledowski et al. (2004a,b) separately applied fMRI and EEG in visual oddball P300 tasks and suggested involvement of the parietal and occipital areas. In these previous studies, distractor stimuli and pseudo-random or relatively long ISI were used to elicit the P300 response. The current study applied a fixed and short ISI, because these are usually used in visual P300-BCI paradigms to facilitate rapid communication (Sellers et al., 2006; Takano et al., 2009b, 2011; Townsend et al., 2010; Ikegami et al., 2011; Pires et al., 2011), but the activated foci observed under both the white/gray and green/blue conditions overlapped with former P300 neuroimaging studies (Clark et al., 2000; Bledowski et al., 2004a,b).

The important roles of the parietal and occipital channels for driving the P300-BCI have been reported repeatedly (Krusienski et al., 2008; Rakotomamonjy and Guigue, 2008; Cecotti et al., 2011). The importance of the parietal and occipital electrodes for improving the performance of the P300-BCI has also been reported (Bianchi et al., 2010; Brunner et al., 2010; Treder and Blankertz, 2010). The parietal and occipital areas are included in the conventional areas for P300s (Clark et al., 2000; Bledowski et al., 2004a,b). A few studies have investigated additional neuronal responses besides P300 in ERP-based BCI studies, in which the early components of visual evoked potentials influence the visual P300 speller performance with or without gaze (Brunner et al., 2010; Treder and Blankertz, 2010). Those studies revealed that the conventional Donchin speller not only depends on the P300 evoked potential (late component) but also on visual evoked potentials (early component) with eye gaze. Our ERP results showed the chromatic change, which was added to luminance change, enhanced the parietal and occipital areas in the late ERP component. Therefore, our P300-BCI paradigms are suggested to be utilizing the brain areas for so-called P300s, rather than other phenomena.

### Methodological considerations

The neuronal mechanisms underlying BCI performance are beginning to be investigated. Recently, Halder et al. (2011) applied fMRI during SMR BCI and suggested the importance of the SMA and the right middle frontal gyrus. EEG is a technology that can be easily applied in real-life situations, but it does not provide the fine spatial resolution necessary for functional investigations; therefore, the addition of fMRI data was useful. In this study, we simultaneously obtained fMRI and EEG recordings. Simultaneous recording of the EEG and fMRI data was enabled by techniques for removing imaging artifacts (Allen et al., 2000; Mandelkow et al., 2006; Goncalves et al., 2007; Grouiller et al., 2007); however, despite much effort, residual artifacts, especially the gradient artifacts, were still very severe in the EEG data acquired during fMRI scanning, so we simply evaluated the evoked response offline. The EEG–fMRI data were still beneficial in that they were provided from the exact same brain areas with the same tasks, which cannot be guaranteed without simultaneous recordings.

In summary, a peak in the positive wave of the EEG data was detected under both conditions, and the peak amplitudes were larger at the parietal and occipital electrodes especially on the right side, particularly in the late components, under the green/blue condition than under the white/gray condition. The fMRI data showed activation in the bilateral parietal and occipital cortices, and these areas, particularly those in the right hemisphere, were more activated under the green/blue condition than under the white/gray condition. Further investigations (for example, of the functional roles of the parietal cortex) are necessary to fully understand the neural basis of the parietal cortex, but our results suggest that the green/blue flicker matrix is useful for enhancing conventional P300 responses.

## Conflict of Interest Statement

The authors declare that the research was conducted in the absence of any commercial or financial relationships that could be construed as a potential conflict of interest.
